# Still a Host of Hosts for *Wolbachia*: Analysis of Recent Data Suggests That 40% of Terrestrial Arthropod Species Are Infected

**DOI:** 10.1371/journal.pone.0038544

**Published:** 2012-06-07

**Authors:** Roman Zug, Peter Hammerstein

**Affiliations:** Institute for Theoretical Biology, Humboldt-Universität zu Berlin, Berlin, Germany; University of Poitiers, France

## Abstract

*Wolbachia* are intracellular bacteria that manipulate the reproduction of their arthropod hosts in remarkable ways. They are predominantly transmitted vertically from mother to offspring but also occasionally horizontally between species. In doing so, they infect a huge range of arthropod species worldwide. Recently, a statistical analysis estimated the infection frequency of *Wolbachia* among arthropod hosts to be 66%. At the same time, the authors of this analysis highlighted some weaknesses of the underlying data and concluded that in order to improve the estimate, a larger number of individuals per species should be assayed and species be chosen more randomly. Here we apply the statistical approach to a more appropriate data set from a recent survey that tested both a broad range of species and a sufficient number of individuals per species. Indeed, we find a substantially different infection frequency: We now estimate the proportion of *Wolbachia*-infected species to be around 40% which is lower than the previous estimate but still points to a surprisingly high number of arthropods harboring the bacteria. Notwithstanding this difference, we confirm the previous result that, within a given species, typically most or only a few individuals are infected. Moreover, we extend our analysis to include several reproductive parasites other than *Wolbachia* that were also screened for in the aforementioned empirical survey. For these symbionts we find a large variation in estimated infection frequencies and corroborate the finding that *Wolbachia* are the most abundant endosymbionts among arthropod species.

## Introduction

*Wolbachia* are the predominant bacterial endosymbionts of arthropods, infecting a vast number of host species worldwide [1]. Both the proportion of infected individuals within species (prevalence) and the overall percentage of infected species (incidence) are important parameters describing the infection frequency of *Wolbachia*. In order to estimate these parameters, Hilgenboecker et al. [2] recently presented a meta-analysis that combined the data from 20 *Wolbachia* screenings with more than 900 arthropod species in total. Using a statistical approach, i.e. a beta-binomial model, they found that prevalences are typically very low or very high, and estimated the incidence of *Wolbachia* to be around 66%, which is considerably higher than previous estimates of approximately 20% [3], [4]. A major reason for such underestimation is the sampling of only one or a few individuals per species. With these one-individual samples, low (and even high) prevalence infections are likely to be overlooked. On the other hand, Hilgenboecker et al. [2] found that samples comprising more than 100 individuals per species tend to be biased towards infected species, e.g. due to prior knowledge of infection. Although they corrected for the latter bias by excluding particularly large samples, many studies used in their meta-analysis still included quite a lot of one-individual samples and were restricted to specific host taxa (see [2] for details). Therefore, in order to more accurately assess the incidence of *Wolbachia* in arthropod hosts, it is crucial to analyze a data set that comprises a medium number of individuals from randomly chosen species. Here, we apply the approach by Hilgenboecker et al. [2] to data from a recent survey by Duron et al. [5] that meets these requirements more closely. This survey also tested for the presence of several reproductive parasites, which allows us to estimate incidences of other endosymbionts and compare them to that of *Wolbachia*.

## Methods

In the survey by Duron et al. [5], 136 species of terrestrial arthropods (2052 individuals in total) were screened for the presence of seven reproductive parasites: *Wolbachia*, *Arsenophonus*, *Cardinium*, *Flavobacterium*, *Rickettsia*, *Spiroplasma ixodetis* and *S. poulsonii.* Since *Flavobacterium* was never observed, we excluded it from our analysis. In the survey, not more than 40 individuals were sampled per species, and in only 25 of the 136 species tested, less than 10 individuals were sampled (median: 15 individuals per species; mode: 20 individuals per species). This range of sampled individuals should help to avoid the drawbacks of both one-individual samples and the bias associated with extensive sampling. Arthropod species tested encompassed 15 orders and three classes (Insecta, Arachnida, Malacostraca), thus representing a widespread and sufficiently random collection. Taken together, the data from Duron et al. [5] should satisfy the requirements for an improved data set as outlined above.

We again use the framework of a beta-binomial model to estimate symbiont prevalence *q* and incidence *x*. Different species are assumed to exhibit different prevalences, and thus *q* values follow a probability distribution *p(q)*. The incidence *x* is then estimated by integrating the prevalence distribution:
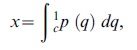
where *c* defines a threshold frequency below which species are considered to be uninfected. For a more detailed account of the model, see [2].

## Results and Discussion

**Figure 1:**
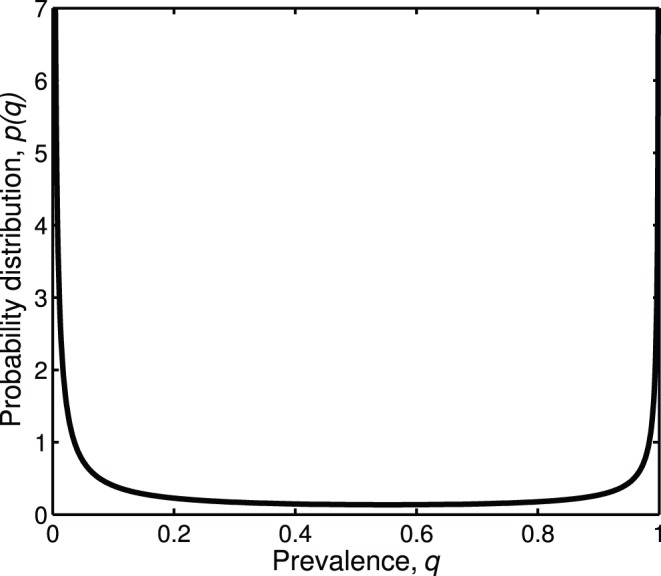
Estimated probability distribution of *Wolbachia* prevalence.

The prevalence distribution for *Wolbachia* shows that either most or only few individuals within a species are infected (Figure 1). Based on this distribution, *Wolbachia* incidence is estimated to be x = 0.406 for c = 0.001 (Table 1). We chose c = 0.001 in accordance with Hilgenboecker et al. [2] to facilitate comparisons. Our results confirm the main qualitative findings from the previous meta-analysis, i.e. the ‘most-or-few’ prevalence pattern and the likely underestimation of incidence in previous *Wolbachia* screenings. However, there is one major difference between the results of the two analyses: In the first study, *Wolbachia* incidence was estimated to be 66% (for c = 0.001). Based on the data from Duron et al. [5], we now obtain a lower estimate of the percentage of *Wolbachia*-infected species, i.e. approximately 40%. We think that our current estimate is more reasonable for the following three reasons.

**Table 1:** Estimates of incidence *x* of different endosymbionts, depending on threshold infection frequency *c*.

Threshold frequency c	Incidence x					
	*Wolbachia*	*Arsenophonus*	*Cardinium*	*Rickettsia*	*Spiroplasma ixodetis*	*S. poulsonii*
0.01	0.335	0.059	0.111	0.014	0.148	0.022
0.001	0.406	0.066	0.162	0.061	0.221	0.032
0.0001	0.470	0.072	0.211	0.114	0.289	0.041

The parameter *c* is the infection frequency above which species are considered infected.

First, the underlying data contain only a very low proportion of species samples in which only a few individuals were tested. Testing only a small number of individuals considerably increases the likelihood of randomly picking some uninfected individuals from an actually infected species, particularly if prevalence levels are low. Indeed, there is evidence that infection frequencies within species are often variable between geographically distinct populations. Such a prevalence variation between populations was found in several species-specific surveys, ranging from 0% to 100% in the cherry fruit fly or from 4% to 100% in two planthoppers [6], [7]. In another fruit fly screening that tested 1500 individuals, only extremely low prevalence levels were found among different populations, ranging from 0% to 3% [8]. Moreover, species might also be falsely classified as uninfected because of low-titer infections that are not detected. Recent evidence suggests that such low-titer *Wolbachia* infections within arthropod hosts are more common than previously thought [7], [9]. Taken together, sampling more than just a few individuals – as it was predominantly done by Duron et al. [5] – avoids the pitfalls outlined above and thus significantly improves estimates of *Wolbachia* infection frequencies in nature.

A second reason why we think our current estimate is more accurate is that the new data set does not include large samples (not more than 40 individuals per species). Large samples are likely to be biased towards infection, probably because respective species were already known to be infected and were sampled extensively to study infection prevalence in more detail. Additionally, large samples will disproportionately often be samples of common species, just because common species are more easily collected in large amounts ([2]; cf. the collecting procedure in [10]). Common species, however, are again prone to have already been tested for infection. These are important issues because large samples inherently have a strong impact on the estimation procedure. Therefore, as was already pointed out by Hilgenboecker et al. [2], omission of large samples will improve estimates of *Wolbachia* incidence.

Thirdly, the fact that Duron et al. [5] sampled a wide range of arthropod species from 15 different orders should render this collection sufficiently diverse in phylogenetic terms. In contrast to the previous meta-analysis which pooled the results from many taxon-specific screenings, analyzing a broad taxon survey ensures that all species are examined with the same detection method. Usually, *Wolbachia* infections are detected by PCR assays which crucially depend on the sensitivity of commonly used PCR primers. A recent assessment of standard PCR protocols used for *Wolbachia* detection, however, reveals considerable variation in primer efficiency [11]. To summarize, the data set compiled by Duron et al. [5] is the first one that satisfies our criteria for a reliable estimate (no one-individual samples, no large samples, no restriction to a specific host taxon). In contrast, several *Wolbachia* screenings that have since been published fail to satisfy the criteria and are not included in our analysis (see, for example, [12]–[16]). We therefore think that our estimate of *Wolbachia* incidence within arthropods is more reliable than previous attempts.

Another reason for the difference in incidence estimates between our study and that by Hilgenboecker et al. [2] might be the different sampling range. As described above, infection frequencies within species can differ greatly between geographically distinct populations. Duron et al. [5] pointed out that such geographical variation in prevalence was likely to increase when expanding the sampling range beyond Western Europe, where species were predominantly collected. In contrast, the meta-analysis by Hilgenboecker et al. [2] comprises samples from most continents, covering both temperate and tropical zones. Although speculative, this unequal geographical sampling might partially explain the difference in incidence estimates derived from both data sets.

Previous broad taxon surveys of *Wolbachia* infection frequencies among arthropods found approximately 20% of the tested species to be infected [3], [4]. In general, previous surveys have estimated incidence by dividing the number of infected species by the overall number of species tested. Adopting the same straightforward approach to the data by Duron et al. [5] yields a very similar estimate (22.8%). However, this is roughly only half of our 40% estimate, although based on the same data set. Therefore, the proportion of *Wolbachia*-infected species seems to be considerably higher than a first glance would suggest.

In order to compare the infection frequency of *Wolbachia* to that of other reproductive parasites, we estimate the incidence for five endosymbionts that were also included in the survey by Duron et al. [5]. Since these symbionts were detected only in very few species, the graphic representation of the prevalence distributions is of limited value and therefore not displayed here. Incidence levels range from 0.032 (*Spiroplasma poulsonii*) to 0.221 (*Spiroplasma ixodetis*; Table 1, all values for c = 0.001). Again, our estimates are consistently higher than those obtained by the straightforward approach (see [5]). Yet, even *S. ixodetis* as the most common of these other symbionts does not match *Wolbachia* in terms of incidence, which corroborates the status of *Wolbachia* as the most abundant reproductive parasite of arthropod hosts. Considering the species richness of the global arthropod community [17], our estimate implies that more than a million species are infected with *Wolbachia*. Thus, although lower than estimated by Hilgenboecker et al. [2], the number of species harboring *Wolbachia* is still remarkably high and justifies further efforts to investigate interactions between these endosymbionts and their arthropod hosts.
